# Community-based adherence clubs for postpartum women on antiretroviral therapy (ART) in Cape Town, South Africa: a pilot study

**DOI:** 10.1186/s12913-020-05470-5

**Published:** 2020-07-08

**Authors:** Allison Zerbe, Kirsty Brittain, Tamsin K. Phillips, Victoria O. Iyun, Joanna Allerton, Andile Nofemela, Cathy D. Kalombo, Landon Myer, Elaine J. Abrams

**Affiliations:** 1 ICAP, Mailman School of Public Health, Columbia University, 722 W. 168th street, 13th floor, New York, 10032 USA; 2 Centre for Infectious Disease Epidemiology & Research, School of Public Health & Family Medicine, University of Cape Town, Cape Town, South Africa; 3 Division of Epidemiology & Biostatistics, School of Public Health & Family Medicine, University of Cape Town, Cape Town, South Africa; 4 Provincial Government of the Western Cape, Cape Town, South Africa; 5 Vagelos College of Physicians & Surgeons, Columbia University, New York, USA

**Keywords:** HIV, Differentiated care, Postpartum, Retention in care, Adherence clubs, Maternal and child health

## Abstract

**Background:**

With an increasing number of countries implementing Option B+ guidelines of lifelong antiretroviral therapy (ART) for all pregnant and breastfeeding women, there is urgent need to identify effective approaches for retaining this growing and highly vulnerable population in ART care.

**Methods:**

Newly postpartum, breastfeeding women who initiated ART in pregnancy and met eligibility criteria were enrolled, and offered the choice of two options for postpartum ART care: (i) referral to existing network of community-based adherence clubs or (ii) referral to local primary health care clinic (PHC). Women were followed at study measurement visits conducted separately from either service. Primary outcome was a composite endpoint of retention in ART services and viral suppression [VS < 50 copies/mL based on viral load (VL) testing at measurement visits] at 12 months postpartum. Outcomes were compared across postpartum services using chi-square, Fisher’s exact tests and Poisson regression models. The primary outcome was compared across services where women were receiving care at 12 months postpartum in exploratory analyses.

**Results:**

Between February and September 2015, 129 women (median age: 28.9 years; median time postpartum: 10 days) were enrolled with 65% opting to receive postpartum HIV care through an adherence club. Among 110 women retained at study measurement visits, 91 (83%) achieved the composite endpoint, with no difference between those who originally chose clubs versus those who chose PHC services. Movement from an adherence club to PHC services was common: 31% of women who originally chose clubs and were engaged in care at 12 months postpartum were attending a PHC service. Further, levels of VS differed significantly by where women were accessing ART care at 12 months postpartum, regardless of initial choice: 98% of women receiving care in an adherence club and 76% receiving care at PHC had VS < 50 copies/mL at 12 months postpartum (*p* = 0.001).

**Conclusion:**

This study found comparable outcomes related to retention and VS at 12 months postpartum between women choosing adherence clubs and those choosing PHC. However, movement between postpartum services among those who originally chose adherence clubs was common, with poorer VS outcomes among women leaving clubs and returning to PHC services.

**Trial registration:**

ClinicalTrials.gov NCT02417675, April 16, 2015 (retrospectively registered).

## Background

Shifting guidelines away from CD4-guided antiretroviral therapy (ART) eligibility (Option A) to universal ART for HIV-infected pregnant and postpartum women (Option B+) has led to a significant increase in the number of women on ART during pregnancy and breastfeeding [1]. While the Option B+ approach offers substantial benefits for the prevention of mother-to-child transmission (PMTCT) and maternal and child health (MCH), concerns related to suboptimal maternal ART adherence and retention in HIV care have been well-documented [2–5]. While a study comparing CD4-guided ART eligibility to Option B+ in Eswatini did find improved retention at 6 months postpartum among women accessing PMTCT services under Option B+, the authors noted that retention, specifically postnatally, was low across both approaches [6]. In a recent meta-analysis, retention rates among women enrolled in Option B+ programs were found to be below those of the general adult population engaged in ART care [7]. Further, all studies included reported increasing loss to follow-up over time, highlighting specific concerns related to retention beyond the immediate postpartum period. With an increasing number of countries implementing the Option B+ strategy and in turn managing a growing number of pregnant and postpartum women on ART, there is an urgent and critical need to identify effective approaches to retaining this highly vulnerable population.

Outside of MCH services, exciting and innovative ART service delivery models have been rolled out, specifically for stable adult patients established on ART [8–10]. Endorsed by the World Health Organization (WHO) in their 2016 guidelines [11], these cost-effective differentiated service delivery (DSD) models may lead to higher rates of retention and adherence among stable patients, while decongesting overburdened health systems [12–16]. In Cape Town, South Africa, a network of community-based adherence clubs has evolved since 2012 and now offers HIV care to over 32,000 ART patients (25% of all ART patients) across the Cape Town health district [17, 18]. To be eligible for the club system, patients must be non-pregnant adults, on ART for 6–12 months, with a suppressed viral load (VL) and no adherence issues or comorbidities that require ongoing clinical management. In this setting, high levels of retention and viral suppression among adults who are attending clubs have been reported [18].

Despite progress in rolling out various DSD models in Cape Town and other locations, questions remain about their potential among pregnant and postpartum women. To date, these models, including adherence clubs in the Cape Town health district, have focused primarily on stable patients established on ART with a suppressed VL and have actively excluded other key and vulnerable populations including pregnant and breastfeeding women [19]. However, recent advocacy by the WHO and others has encouraged expansion of these models to pregnant and postpartum women, recognizing that with the roll-out of Option B+, there are increasing numbers of pregnant women presenting for antenatal care with known HIV status and who are stable on ART as well as large groups of women initiating ART in pregnancy [19, 20]. With this in mind, we conducted a pilot evaluation of the community-based adherence club model among newly postpartum women who had initiated ART during their recent pregnancy in Cape Town, South Africa. Our aim was to explore uptake of the adherence club model during the postpartum period, as well as programmatic outcomes among women choosing to attend these clubs.

## Methods

### Design and setting

Conducted between February 2015 and October 2016, the Postpartum Adherence Clubs to Enhance Support (PACER) study (ClinicalTrials.gov NCT02417675) was a component of the MCH-ART study (NCT01933477), a multicomponent implementation science study evaluating strategies for delivering HIV care and treatment services during pregnancy and the postpartum period [21]. The aim of the PACER study was to pilot and evaluate the enrollment of postpartum women into a network of adherence clubs for receipt of ART care. The study took place around the Midwife-Obstetric Unit of the Gugulethu Community Health Centre, a setting characterized by high levels of poverty and a high antenatal HIV prevalence [21]. In this setting, Option B+ guidelines were rolled out during 2013. All participants provided written informed consent prior to enrollment, and the study was approved by the Human Research Ethics Committee of the University of Cape Town’s Faculty of Health Sciences as well as the Institutional Review Board of the Columbia University Medical Center.

### Parent MCH-ART study

The design and results of the parent MCH-ART study have been previously described [21, 22]. The study included a randomized trial of postpartum ART services, comparing (i) an integrated postnatal care service within the MCH setting for HIV-infected mothers and their infants for the duration of breastfeeding to (ii) the local standard of care, immediate referral to adult ART services for mothers and separate ‘well baby’ services for infants. The primary outcome was a combined endpoint of maternal engagement in HIV care and viral suppression (VS) < 50 copies/mL at 12 months postpartum.

### PACER study methods

The methods of the PACER study parallel those of the MCH-ART study, although separate cohorts were enrolled for each study. The PACER study methods and preliminary findings through 6 months postpartum have been reported previously [23]. Briefly, HIV-infected recently postpartum women who were > 18 years old, had initiated ART during their recent pregnancy, intended to stay in the area through 12 months postpartum and were currently breastfeeding were eligible. In addition, women had to meet eligibility requirements for referral to the adherence club system: documented VS < 1000 copies/mL (based on VL at 12 weeks post ART initiation) and no comorbidities requiring on-going clinical review. Once enrolled into the PACER study, participants were given a choice between two options for their postpartum ART care: (i) referral to existing network of community-based adherence clubs or (ii) referral to a local primary health care clinic (PHC). Following their choice, women were referred to their chosen service using standard referral procedures.

### Postpartum care services

Antenatal services and the postpartum care services to which participants were referred have been previously described [23]. In brief, postpartum women were referred < 2 weeks post-delivery from integrated antenatal ART/PMTCT services to a network of PHCs under the standard of care. PHCs provided general adult ART services at visits conducted every 1–2 months that included ART collection and a clinical consultation with a doctor or nurse. HIV-exposed infants were referred separately for routine ‘well baby’ services including early infant diagnosis (EID).

Community-based adherence clubs were facilitated by lay counselors and housed within community venues separate from public sector health facilities. Clubs met every 2–4 months at which time patients were weighed, completed a brief symptom questionnaire and participated in health education sessions. Pre-packed ART was dispensed at all visits to patients or “treatment buddies” identified by the patient. Clinical review and VL testing were conducted annually by a nurse. Patients were referred back to routine PHC if they missed a scheduled club visit, had a detectable VL or comorbidity requiring clinical management, based on the standard criteria for continued attendance at clubs in this setting.

### Study follow-up & sources of data

Regardless of their choice of postpartum service, all women enrolled in the PACER study were followed at repeated study measurement visits that were scheduled and located separately from any club or PHC care visits through 12 months postpartum. These study measurement visits were held within a dedicated research center and conducted by trained study staff. At each study visit, questionnaires related to demographics, MCH service utilization, ART adherence, and breastfeeding practices were administered. Questions regarding ART service experience were asked at 9 months postpartum. Additionally, women provided 5 ml of venous blood at each visit for batched HIV RNA VL testing by the South African National Health Laboratory Services (NHLS) using the Abbott RealTime HIV-1 assay (Abbott Laboratories, Abbott Park, Illinois, US).

Data on maternal and infant use of healthcare services were drawn from routinely collected public sector medical records (including facility registers, electronic medical records and infants’ Road to Health Booklets) and centralized NHLS databases of laboratory tests, abstracted for all participants at the end of the study period.

### Outcomes

The primary study outcome was a composite endpoint of women’s retention in postpartum ART services and VS < 50 copies/mL (based on VL testing at measurement visits) at 12 months postpartum. Women were required to be both retained in care and virally suppressed in order to be considered as having achieved the primary outcome. Retention in care at 12 months postpartum was measured using routinely collected medical records and defined as evidence of an HIV-related clinical contact (whether from HIV-related laboratory testing or clinical care/adherence club visit) from 9 to 18 months postpartum. Where women had evidence of multiple clinical contacts from 9 to 18 months postpartum, we used the contact closest to 12 months postpartum to classify women as retained in a PHC service versus retained in an adherence club. Secondary outcomes included duration of any breastfeeding and exclusive breastfeeding (EBF) (self-reported at all study visits), and infant engagement outcomes (evidence of PCR testing for EID and immunizations) abstracted from routinely- collected medical records. Because outcome data on retention in postpartum ART care came from medical records available from all facilities in the province and did not require separate study follow-up, this outcome was available for all participants (regardless of completion of study visits and availability of VL outcome data, or movement out of Gugulethu) and is presented for all women enrolled as well as restricted to women who had VL outcome data.

### Analysis

Data were analysed using Stata 12 (StataCorp Inc., College Station, Texas, USA). Variables were described using medians (with interquartile ranges, IQR) and proportions. We examined factors associated with retention at study measurement visits (defined as attending a study visit > 270 days postpartum) using Wilcoxon rank-sum (Mann Whitney) and chi-square or Fisher’s exact tests in the case of sparse data. We compared primary and secondary study outcomes across postpartum ART service choice using chi-square and Fisher’s exact tests, and used product-limit methods and the log-rank test to compare duration of breastfeeding and EBF across postpartum ART service choice. For the primary outcome, we defined VS as < 50 copies/mL, but examined VS < 1000 copies/mL in sensitivity analyses. In additional sensitivity analyses, we explored the effect of loss to follow-up by assuming that women who were not retained at study measurement visits had (i) suppressed and (ii) elevated VL, respectively. In order to account for potential confounding, we examined the independent effect of choice of ART service on the primary outcome in Poisson regression models with robust error variance. Given that our intention was to assess programmatic outcomes among women choosing to attend adherence clubs, we additionally explored referral out of clubs and compared the primary outcome across the service where women were receiving care at 12 months postpartum.

## Results

### Study enrollment and retention

A total of 129 women (median age: 28.9 years; median time postpartum: 10 days) were enrolled between February and September 2015 (Table 1). Women had been on ART for a median time of 23.3 weeks at enrollment, 80% had experienced a previous pregnancy, and most (59%) were newly diagnosed HIV-positive during their recent pregnancy. A total of 84 women (65%) opted to receive postpartum HIV care through an adherence club, with no differences in demographic or clinical characteristics among women choosing adherence clubs versus those choosing PHC. A total of 110 women (85%) were retained at study measurement visits through 270 days postpartum, with a median time at outcome assessment of 12.1 months postpartum (IQR: 12.0–12.3 months). Compared to women retained at study measurement visits, women lost to follow-up were significantly more likely to have opted to receive postpartum HIV care from a PHC (63% versus 37%; *p* = 0.005).

**Table 1:** Demographic and clinical characteristics of participants at enrolment, stratified by retention at study visits

Variable	Total sample – *n* (%)	Completed study visit > 270 days postpartum – *n* (%)	Lost to study ≤270 days postpartum – *n* (%)	*p*-value
Number of women	129	110	19	
ART service choice				
Adherence club	84 (65)	77 (70)	7 (37)	
Primary care clinic	45 (35)	33 (30)	12 (63)	0.005
Median [IQR] age	28.9 [24.5, 32.1]	28.8 [24.3, 32.3]	28.9 [25.6, 31.2]	0.984
First pregnancy	26 (20)	24 (22)	2 (11)	0.360
Completed secondary/any tertiary education	60 (47)	53 (48)	7 (37)	0.360
Currently employed	45 (35)	40 (36)	5 (26)	0.396
Married/cohabiting	48 (37)	41 (37)	7 (37)	0.971
Newly diagnosed HIV+ in this pregnancy	76 (59)	67 (61)	9 (47)	0.268
Pregnancy intention				
Unintended	88 (68)	74 (67)	14 (74)	0.790
Intended	41 (32)	36 (33)	5 (26)	
Median [IQR] CD4 cell count				
≤ 350 cells/μL	56 (43)	48 (44)	8 (42)	
> 350 cells/μL	73 (57)	62 (56)	11 (58)	0.901
HIV viral load				
< 50 copies/mL	114 (93)	99 (94)	15 (83)	
≥ 50 copies/mL	9 (7)	6 (6)	3 (17)	0.126
Median [IQR] time on ART (weeks)	23.3 [18.1, 26.9]	23.7 [18.4, 27.0]	21.0 [15.6, 26.7]	0.266
Median [IQR] days postpartum	10 [5, 19]	10 [6, 19]	8 [5, 15]	0.180
Place of delivery				
Primary care	50 (39)	41 (37)	9 (47)	
Hospital care	79 (61)	69 (63)	10 (53)	0.404
Reported missed ART dose(s) during previous 30 days	19 (15)	18 (16)	1 (5)	0.304
Exclusively breastfed infant up to enrolment	106 (82)	88 (80)	18 (95)	0.193

### Comparison of primary outcome across ART services

Among the 110 women retained at study measurement visits, 91 (83%) achieved the composite endpoint of engagement in care and VS at 12 months postpartum, with no difference between those who originally chose adherence clubs versus those who chose PHC services (84% versus 79%, respectively; *p* = 0.583; Table 2). Within this composite outcome, 87% of women (*n* = 96) had evidence of retention in ART services at 12 months postpartum, with retention in care not significantly different by choice of ART service: 90% of those who originally chose club services were retained in care, compared to 82% of those who chose PHC services (*p* = 0.349). Among all participants enrolled (*n* = 129), retention at study measurement visits was not associated with retention in care (*p* = 0.304). In this total sample, retention in care outcomes were similar across choice of ART services: 88% of women who chose club services were retained in care, compared to 82% of women who chose PHC services (*p* = 0.426). Similarly, no difference was observed in VS by ART service under various definitions of viral suppression (Table 2). For example, 90% of women who chose adherence club services and 85% of women who chose PHC services had VS < 1000 copies/mL at 12 months postpartum (*p* = 0.525). After adjustment for maternal age and duration on ART, the lack of association between original choice of postpartum ART service and VS < 50 copies/mL at 12 months postpartum persisted (risk ratio for adherence club versus PHC: 1.08; 95% confidence interval: 0.88, 1.31).

**Table 2:** Comparison of composite endpoint and uptake of infant care services by ART service choice

Variable	Total sample – *n* (%)	Adherence club – *n* (%)	Primary care clinic – *n* (%)	*p*-value
***Composite endpoint: Evidence of maternal retention in HIV care AND VL < 50 copies/mL at 12 months postpartum***				
Number of women eligible for composite endpoint	110	77	33	
N (%) achieving composite endpoint	91 (83)	65 (84)	26 (79)	0.583
***Retention in care (in all participants retained at study visits with VL data available)***				
Number of women retained at study visits with VL data available	110	77	33	
Evidence of engagement in HIV care at 12 months postpartum	96 (87)	69 (90)	27 (82)	0.349
***Retention in care (in all participants enrolled)***				
Number of women enrolled	129	84	45	
Evidence of engagement in HIV care at 12 months postpartum	111 (86)	74 (88)	37 (82)	0.426
***Viral load (in all participants retained at study visits with VL data available)***				
Number of women retained at study visits with VL data available	110	77	33	
VL < 50 copies/mL at 12 months postpartum	91 (83)	65 (84)	26 (79)	0.583
VL < 1000 copies/mL at 12 months postpartum	97 (88)	69 (90)	28 (85)	0.525
***Infant care services***				
Number of women	129	84	45	
Evidence of infant PCR test < 12 weeks of age	123 (95)	81 (96)	42 (93)	0.420
Number of women retained in the study with infant immunization records available	106	75	31	
Evidence of birth immunizations	104 (98)	73 (97)	31 (100)	1.000
Evidence of 6 week immunizations	99 (93)	68 (91)	31 (100)	0.103
Evidence of 10 week immunizations	97 (92)	68 (91)	29 (94)	1.000

*VL* Viral load

Given loss to follow-up from the study and assuming that women who were not retained at study measurement visits had (i) suppressed and (ii) elevated VL, respectively, the proportion of women with VS < 50 copies/mL at 12 months postpartum in the total sample ranged between 71 and 85%. Under the assumption that all women lost from study measurement visits had suppressed VL < 50 copies/mL, no difference between those who originally chose adherence clubs versus those who chose PHC services was observed (86% versus 84%, respectively; *p* = 1.000). However, when all women lost to follow-up were assumed to have elevated VL, those who originally chose adherence clubs were significantly more likely to have suppressed VL compared to those who chose PHC services (77% versus 58%, respectively; *p* = 0.026).

### Comparison of secondary outcomes across ART services

**Fig. 1:**
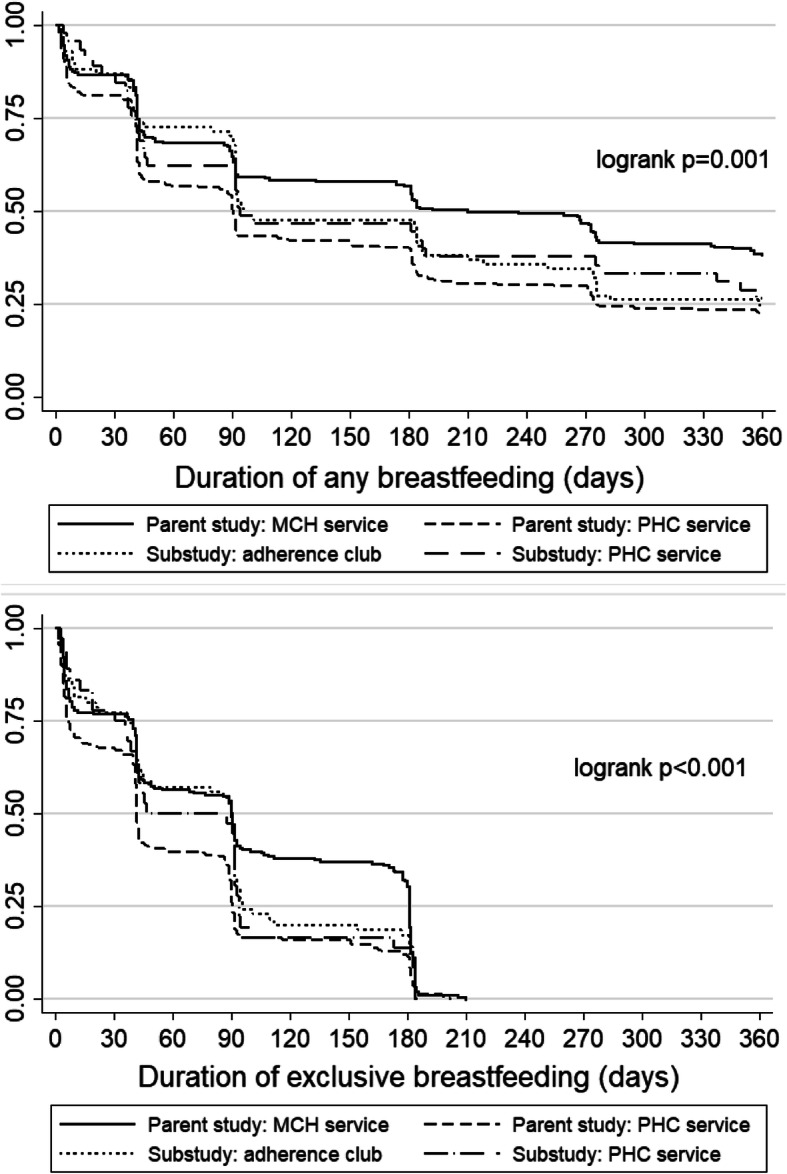
**a** Time to cessation of any breastfeeding, stratified by postpartum ART service [maternal/child health (MCH) service, primary health care (PHC) service, or adherence club]. **b** Time to cessation of exclusive breastfeeding, stratified by postpartum ART service [maternal/child health (MCH) service, primary health care (PHC) service, or adherence club]

The median duration of any breastfeeding was 3.2 months [IQR: 1.4–11.8 months] and did not differ by choice of postpartum ART service (Fig. 1a). Among 106 women who reported EBF at enrolment, the median duration of EBF was 3.0 months [IQR: 1.2–3.1 months (Fig. 1b). Uptake of infant care services was high in both groups. Overall, 95% of mothers had evidence of infant PCR testing between birth and 12 weeks of age (Table 2). Among 106 women who were retained in the study with infant medical records available, uptake of infant immunization through 10 weeks of infant age was similarly high. Neither breastfeeding nor uptake of infant care services differed across ART service choice.

### Experiences in postpartum ART services

Of the 110 women retained at study measurement visits, 102 completed the ART service experience assessment at 9 months postpartum. Women attending an adherence club were positive about these services, with 98% reporting that services were “excellent” or “very good” (Table 3). Participants cited support from peers and counsellors, less frequent and shorter visits, and receiving care outside of health facilities as aspects of club services that they liked. In contrast, women attending PHC services reported mixed feelings about these services, with 65% reporting that the services were “excellent” or “very good”. These women were also less positive about the frequency and duration of visits, although 78% reported that they preferred to receive care within a health facility.

**Table 3:** Experiences in ART services (*n* = 102)

Adherence club (*n* = 65)	*n* (%)	Primary care clinic (PHC; *n* = 37)	*n* (%)
Number of visits at adherence club		Number of visits at PHC^a^	
1	6 (9)	1	2 (5)
2	27 (42)	2	12 (32)
3 or more	32 (49)	3 or more	22 (59)
Prefers that club is closer to where she lives compared to PHC	47 (72)	Prefers that PHC is closer to where she lives compared to club	24 (67)
Likes that club is close to where her baby receives healthcare	45 (69)	Likes that PHC is close to where her baby receives healthcare	24 (65)
Prefers to receive care outside of health facility	61 (94)	Prefers to receive care within health facility	29 (78)
Likes that there are only 5 visits per year	63 (97)	Likes having visits every 4–8 weeks	21 (58)
Likes that the visits are 1 h or less	62 (95)	Likes the amount of time spent at the PHC and pharmacy	16 (44)
Likes not seeing a doctor/nurse frequently	51 (78)	Likes seeing a doctor/nurse frequently	25 (68)
Likes receiving peer support from club group	64 (98)	–	–
Likes receiving care and support from a counsellor	64 (98)	–	–
Overall feelings about club		Overall feelings about PHC	
Excellent	38 (58)	Excellent	9 (24)
Very good	26 (40)	Very good	15 (41)
Good	1 (2)	Good	6 (16)
Bad	0 (0)	Bad	4 (11)
Very bad	0 (0)	Very bad	3 (8)

^a^Missing data from one participant

### Comparison with the parent MCH-ART study

A total of 471 women were enrolled into the randomized trial of the parent MCH-ART study, with women randomized to the integrated MCH-focused postpartum care intervention having significantly higher levels of retention and VS through 12 months postpartum compared to women randomized to PHC services [22]. When comparing the PHC arms of the parent MCH-ART and the PACER study, we observed higher levels of engagement in care and VS at 12 months postpartum among women accessing standard of care PHC services in the PACER study: 79% of PACER participants choosing PHC services achieved the composite endpoint versus 56% of participants randomized to PHC services in the parent study. However, levels of engagement and VS were higher among women randomized to the MCH-focused service in the parent study (84%) versus either the MCH-ART or PACER PHC arm. Duration of breastfeeding was similar across women choosing club services and those attending PHC services in both cohorts, but was significantly longer in women attending the MCH-focused service in the parent study (Fig. 1a and b). No appreciable differences in the uptake of infant care services were observed across any of the ART services.

### Movement between postpartum ART services

**Fig. 2:**
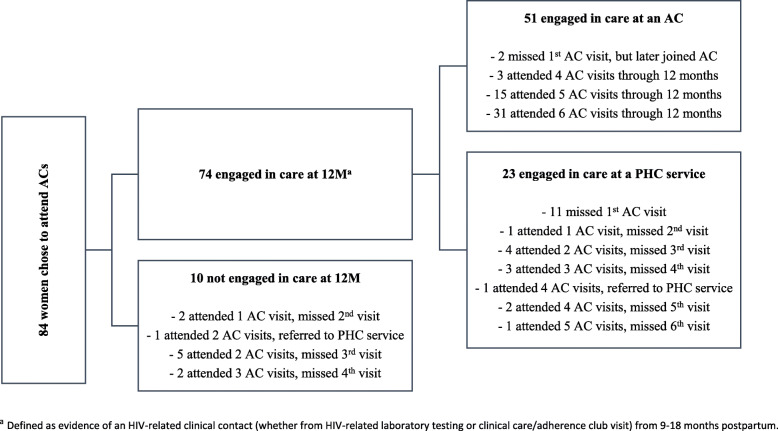
Movement between service models among women originally choosing adherence clubs

Movement from adherence clubs to PHC services is described in Fig. 2. Of the 84 women who originally chose to attend adherence club services, 13 (15%) were found to have never attended their first club meeting [21]. On further medical record review, 12 of these women were found to have immediately accessed care at a PHC after postpartum referral, and 2 later joined an adherence club. Among the 74 women who originally chose to attend club services and were engaged in care at 12 months postpartum, 31% (*n* = 23) were found to be engaged at a PHC service; 2 women (5%) who originally chose PHC services and were engaged in care at 12 months postpartum had evidence of engagement at an adherence club.

**Fig. 3:**
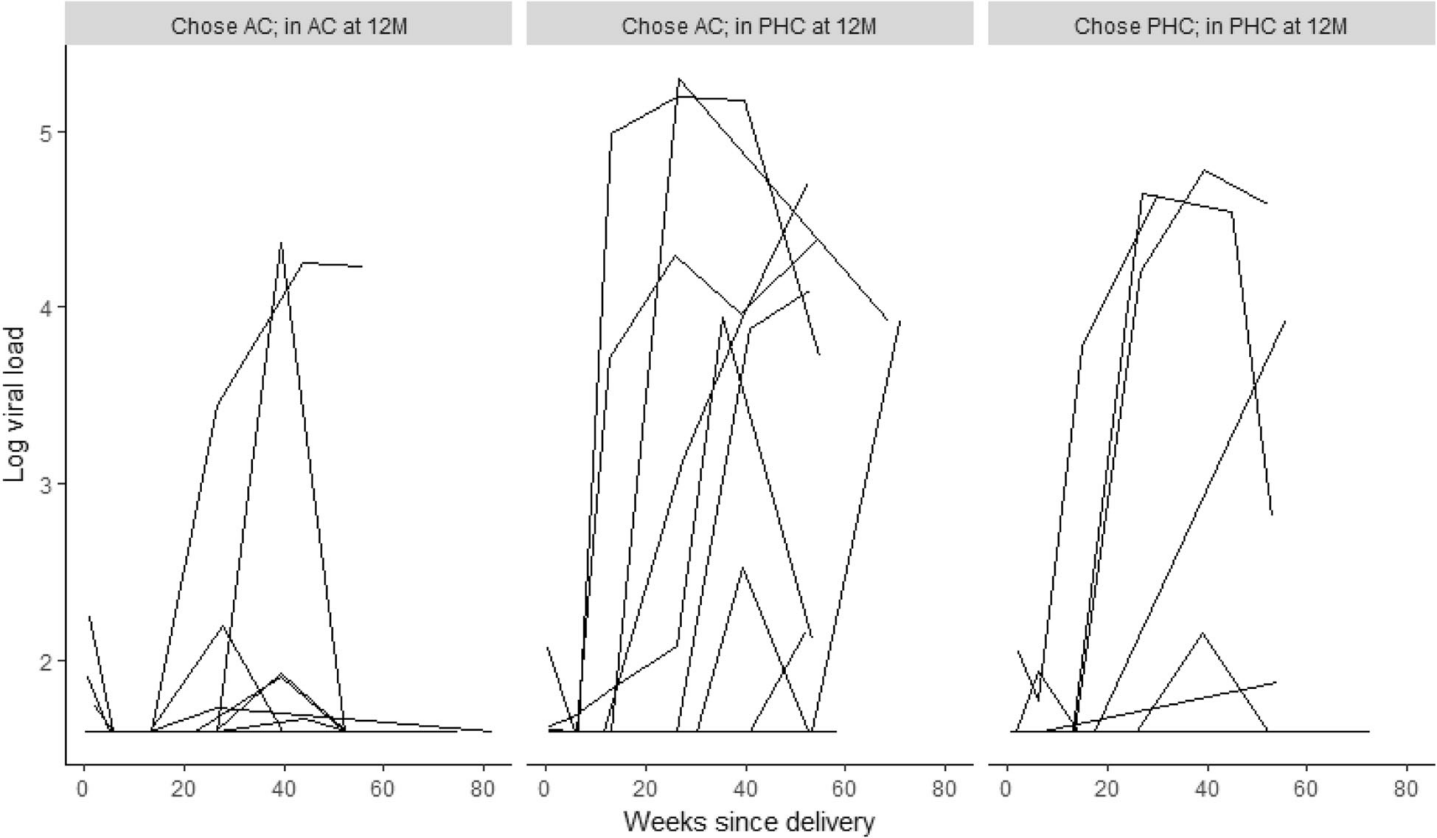
Spaghetti plots of viral load measures conducted at study measurement visits, from delivery through approximately 12 months postpartum, with each line representing a participant and stratified by women who 1) originally chose to attend adherence clubs (AC) and remained in AC at 12 months postpartum (left-hand panel), 2) originally chose to attend AC but moved to primary health care (PHC) services by 12 months postpartum (center panel) and 3) originally chose to attend PHC and remained in PHC at 12 month postpartum

Given high levels of movement from adherence clubs back to PHC services, we explored VS outcomes by the location of ART care. Among the 96 women who had evidence of retention in ART services at 12 months postpartum and VL data available, levels of VS differed significantly across the location of ART care: 98% of those receiving care in an adherence club and 76% of women receiving care at PHC services had VS < 50 copies/mL at 12 months postpartum (*p* = 0.001), regardless of initial choice of postpartum ART services. The results of VL testing at study measurement visits are shown in Fig. 3, by original choice of ART service and location of ART care at 12 months postpartum. Overall, women who chose to attend adherence clubs and remained in these services through 12 months postpartum were less likely to experience elevated VL compared to women who chose to attend adherence clubs but later moved to PHC services, or women who chose to attend PHC services and stayed in these services through 12 months postpartum.

## Discussion

In this study of participation in adherence clubs among newly postpartum women, we found comparable outcomes related to retention in care and VS at 12 months postpartum between women choosing adherence clubs and those choosing PHC services. These data provide longer-term virologic outcomes that are consistent with and compliment the short-term (6-months postpartum) VS outcomes previously reported [23]. While the earlier short-term outcomes captured the immediate postnatal period- a critical time in the PMTCT cascade- these longer-term outcomes are important given the well-documented challenges around non-adherence and disengagement from care among this group [7]. Despite the high overall levels of engagement and VS observed here, we note substantial movement out of adherence clubs and significantly poorer VS outcomes among women who leave adherence clubs and return to PHC services. Regarding secondary outcomes, postpartum women attending adherence clubs were comparable to those attending PHCs in terms of duration of breastfeeding and uptake of infant care services.

Women who chose and attended clubs reported greater satisfaction with these services than those who opted for and attended PHCs. Despite this, we found a significant amount of movement between postpartum services among those who originally chose adherence clubs. By the end of the study follow-up period, one third of those who initially chose and attended the clubs and were engaged in care at 12 months postpartum were found to be attending a PHC. The finding that these women have significantly poorer VS outcomes compared to those who originally chose and remained in adherence clubs suggests that women who remain in clubs do well in this service and that those who leave the service (either by choice or referral back due to clinical indication) and return to PHC services have poorer VS outcomes, despite remaining engaged in care.

These data also highlight the complexities of monitoring and evaluating the impact of adherence clubs and DSD models, generally. We have previously discussed how evaluations that begin with patients making their first visit to an adherence club likely overestimate retention, given the high proportion of women who did not attend their first adherence club visit [23]. Here, we demonstrate that a cross-sectional analysis of women attending adherence clubs at 12 months postpartum does not consider those who have dropped out of clubs or who were referred back to PHC services and who by definition have poor outcomes. As women who newly initiated ART during pregnancy, none of the participants had experience with either postpartum service at the time that they were asked to choose a location for their postpartum care. It is possible that upon attending their chosen service, other factors (geography, fear of stigma, desire for more clinical oversight) emerged and influenced their decision to remain in the club or to seek care elsewhere [24, 25]. High levels of mobility among postpartum women, including in South Africa, have been documented and may also play a role in patterns of clinic movement [26, 27]. There are specific vulnerabilities associated with postpartum transition from one service delivery model to another and increased risk associated with this transfer process [25]. Interventions to facilitate and monitor the safe transition from one model or site to another is an issue that requires further attention as this DSD model is expanded.

The duration of breastfeeding here was comparable among postpartum women attending clubs and the PHC arm of both the parent MCH-ART study and PACER study but was significantly longer in women attending the MCH-focused service in the parent study. Unlike the intervention arm of the MCH-ART study, women in the PACER study were referred to a network of existing adherence clubs, none of which were tailored or customized to the needs of postpartum women. The need for multilevel support to address the multiple and complex needs of postpartum women and their families during this time is of critical importance. Integration of MCH services including family planning, counselling around infant feeding and HIV PCR testing for infants into clubs specifically for postpartum women is an idea that has not yet been explored fully, may be critical to the success of DSD for this population and should be considered as the AC model expands both in South Arica and other settings. An additional consideration may also include whether these groups could be further tailored for women with known HIV infection and who are stable on ART at the time of entry into ANC services versus those who are newly diagnosed and initiated ART in their most recent pregnancy [28].

These results are subject to several limitations. For this pilot study, women were offered a choice of postpartum ART services. Given that women were not randomly allocated to services, confounding may be a concern, but we observed no differences in demographic or clinical characteristics between women choosing each service, and adjustment for age and duration on ART did not change the results observed. Further, exposure to the intervention (postpartum service) changed over time as women moved from adherence clubs to PHCs. However, it was not possible to accurately parse out periods during which women were in the adherence clubs versus PHCs versus not in care, as not all data systems include scheduled appointment dates. The setting for this study was limited to adult women from one urban area in South Africa and results may not be generalizable to other populations. The study sample included only women who had initiated ART during their recent pregnancy, thus there was little variability in the duration of ART use among these women. However, future studies should evaluate the effectiveness of postpartum adherence clubs for women who are already established on ART when entering antenatal care. Additionally, while this is the first evaluation of its kind, the sample size was relatively small and thus the power to detect quantitative associations is limited. In total, 15% of women were lost from study measurement visits, which is consistent with the amount of loss to follow-up in the parent MCH-ART study (13%) [22]. This study is strengthened by its use of VL measures carried out independently from routine care and a retention measure that was not specific to only the facility of interest and was available for all women regardless of retention at study measurement visits. In addition, outcomes included retention and VS measured over a 12-month period. We observed high levels of retention in care and VS overall, likely due to the fact that all women enrolling into the study were required to meet the eligibility criteria for referral into the adherence club system, including VS < 1000 copies/mL. However, under the assumption that all women lost from study measurement visits had elevated VL at 12 months postpartum, the proportion with VS was lower (71% versus 83% when restricted to women retained in the study).

## Conclusions

In summary, this study suggests comparable outcomes related to retention in care and VS at 12 months postpartum between women choosing adherence clubs and those choosing PHC services. We observed a higher reported degree of satisfaction among women attending adherence clubs, but this did not translate into higher rates of retention in the club, with many women leaving the clubs to return to the standard of care facilities, and higher levels of elevated VL in these women. Ongoing work to understand adherence and retention among postpartum women is needed given the growing number of women on ART in the postpartum period. To optimize implementation of adherence clubs and other DSD models among newly postpartum women, additional work is needed to identify strategies to integrate MCH services into these settings.

## Data Availability

The datasets used and/or analyzed during the current study are available from the corresponding author on reasonable request.

## Abbreviations

ART: Antiretroviral therapy
DSD: Differentiated service delivery
EBF: Exclusive breastfeeding
EID: Early infant diagnosis
IQR: Interquartile range
MCH: Maternal child health
PHC: Primacy health clinic
PMTCT: Prevention of mother-to-child transmission
VL: Viral load
VS: Viral suppression
WHO: World Health Organization
